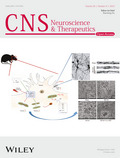# Front cover

**DOI:** 10.1111/cns.14399

**Published:** 2023-08-04

## Abstract

The cover image is based on the Original Article *14, 15‐EET alleviates neurological impairment through maintaining mitochondrial dynamics equilibrium via AMPK/SIRT1/FoxO1 signal pathways in mice with cerebral ischemia reperfusion* by Jing Tang et al., https://doi.org/10.1111/cns.14198.